# Drug resistance phenotypes and genotypes in Mexico in representative gram-negative species: Results from the infivar network

**DOI:** 10.1371/journal.pone.0248614

**Published:** 2021-03-17

**Authors:** Elvira Garza-González, Paola Bocanegra-Ibarias, Miriam Bobadilla-del-Valle, Luis Alfredo Ponce-de-León-Garduño, Verónica Esteban-Kenel, Jesus Silva-Sánchez, Ulises Garza-Ramos, Humberto Barrios-Camacho, Luis Esaú López-Jácome, Claudia A. Colin-Castro, Rafael Franco-Cendejas, Samantha Flores-Treviño, Rayo Morfín-Otero, Fabian Rojas-Larios, Juan Pablo Mena-Ramírez, María Guadalupe Fong-Camargo, Cecilia Teresita Morales-De-la-Peña, Lourdes García-Mendoza, Elena Victoria Choy-Chang, Laura Karina Aviles-Benitez, José Manuel Feliciano-Guzmán, Eduardo López-Gutiérrez, Mariana Gil-Veloz, Juan Manuel Barajas-Magallón, Efren Aguirre-Burciaga, Laura Isabel López-Moreno, Rebeca Thelma Martínez-Villarreal, Jorge Luis Canizales-Oviedo, Carlos Miguel Cetina-Umaña, Daniel Romero-Romero, Fidencio David Bello-Pazos, Nicolás Rogelio Eric Barlandas-Rendón, Joyarib Yanelli Maldonado-Anicacio, Enrique Bolado-Martínez, Mario Galindo-Méndez, Talia Perez-Vicelis, Norma Alavez-Ramírez, Braulio J. Méndez-Sotelo, Juan Francisco Cabriales-Zavala, Yirla Citlali Nava-Pacheco, Martha Irene Moreno-Méndez, Ricardo García-Romo, Aldo Rafael Silva-Gamiño, Ana María Avalos-Aguilera, María Asunción Santiago-Calderón, Maribel López-García, María del Consuelo Velázquez-Acosta, Dulce Isabel Cobos-Canul, María del Rosario Vázquez-Larios, Ana Elizabeth Ortiz-Porcayo, Arely Elizabeth Guerrero-Núñez, Jazmín Valero-Guzmán, Alina Aracely Rosales-García, Heidy Leticia Ostos-Cantú, Adrián Camacho-Ortiz

**Affiliations:** 1 Hospital Universitario Dr. José E. González, Universidad Autónoma de Nuevo León, Monterrey, Nuevo León, Mexico; 2 Instituto Nacional de Ciencias Médicas y Nutrición Salvador Zubirán, Ciudad de México, Mexico; 3 Instituto Nacional de Salud Pública, Cuernavaca, Morelos, Mexico; 4 Instituto Nacional de Rehabilitación Luis Guillermo Ibarra Ibarra, Ciudad de México, Mexico; 5 Hospital Civil de Guadalajara E Instituto de Patología Infecciosa, Guadalajara, Jalisco, Mexico; 6 Hospital Regional Universitario de Colima, Colima, Colima, Mexico; 7 Hospital General de Zona 21 Tepatitlán De Morelos, Centro Universitario de los Altos (CUALTOS), Universidad de Guadalajara, Tepatitlán de Morelos, Jalisco, Mexico; 8 Hospital General Regional No. 1, Chihuahua, Chihuahua, Mexico; 9 Hospital General con Especialidades Juan María de Salvatierra, La Paz, Baja California Sur, Mexico; 10 Hospital Angeles Valle Oriente, Monterrey Nuevo León, Mexico; 11 Hospital General de Zona No. 1, Tapachula, Chiapas, Mexico; 12 Hospital Infantil de Morelia, Morelia, Michoacán, Mexico; 13 Hospital de Especialidades Pediátricas de Chiapas, Tuxtla Gutiérrez, Chiapas, Mexico; 14 Hospital Regional de Alta Especialidad de Oaxaca, San Bartolo Coyotepec, Oaxaca, Mexico; 15 Hospital Regional de Alta Especialidad del Bajío, Guanajuato, Guanajuato, Mexico; 16 Laboratorio Dipromi, Michoacán, Morelia, Mexico; 17 Hospital Regional Delicias, Delicias, Chihuahua, Mexico; 18 Galenia Hospital, Cancún, Quintana Roo, Mexico; 19 Centro Universitario de Salud, Universidad Autónoma de Nuevo León. Laboratorio Vicente Guerrero, Monterrey Nuevo León, Mexico; 20 Centro Universitario de Salud, Universidad Autónoma de Nuevo León. Laboratorio Pueblo Nuevo, Monterrey Nuevo León, Mexico; 21 Hospital Materno Infantil Morelos, Chetumal Quintana Roo, Mexico; 22 Laboratorio de Análisis Bioquímico Clínicos "Louis Pasteur" Toluca, Estado de México, Mexico; 23 Hospital H+ Querétaro, Querétaro, Querétaro, Mexico; 24 Laboratorio Bioclin, Chilpancingo, Guerrero, Mexico; 25 Hospital general de Chilpancingo, Chilpancingo, Guerrero, Mexico; 26 Universidad de Sonora, Hermosillo, Sonora, Mexico; 27 Laboratorios Galindo SC, Oaxaca, Oaxaca, Mexico; 28 Hospital Regional "Bicentenario de la Independencia” ISSSTE, Tultitlán, Estado de México, Mexico; 29 Hospital General “Dr. Manuel Gea González”, Ciudad de México, Mexico; 30 Swiss Hospital, Monterrey Nuevo león, Mexico; 31 Hospital para el Niño Poblano, San Andrés Cholula, Puebla, Mexico; 32 Laboratorios del Centro, Zamnora, Michoacán, Mexico; 33 Centenario Hospital Miguel Hidalgo, Aguascalientes, Aguascalientes, Mexico; 34 Hospital Ángeles Morelia, Morelia, Michoacán, Mexico; 35 Hospital General “Dr. Miguel Silva”, Morelia, Michoacán, Mexico; 36 Hospital General de Zona No 1 Dr. Demetrio Mayoral Pardo, Oaxaca, Oaxaca, Mexico; 37 Hospital de la Madre y Niño Guerrerense, Chilpancingo, Guerrero, Mexico; 38 Instituto Nacional de Cancerología, Ciudad de México, Mexico; 39 Hospital General de Chetumal, Chetumal Quintana Roo, Mexico; 40 Instituto Nacional de Cardiología Ignacio Chávez, Ciudad de México, Mexico; 41 Hospital Regional Monterrey ISSSTE, Monterrey Nuevo León, Mexico; 42 Hospital Militar regional de especialidades, Mazatlán Sinaloa, Mexico; 43 Centro de diagnóstico microbiológico SA de CV, Morelia, Michoacán, Mexico; 44 Hospital de Especialidades pediátrico de León, León, Guanajuato, Mexico; 45 Bioclinsa, Hospital Ginequito, Monterrey Nuevo León, Mexico; University of Nicolaus Copernicus in Torun, POLAND

## Abstract

**Aim:**

This report presents phenotypic and genetic data on the prevalence and characteristics of extended-spectrum β-lactamases (ESBLs) and representative carbapenemases-producing Gram-negative species in Mexico.

**Material and methods:**

A total of 52 centers participated, 43 hospital-based laboratories and 9 external laboratories. The distribution of antimicrobial resistance data for *Escherichia coli*, *Klebsiella pneumoniae*, *Enterobacter cloacae* complex, *Acinetobacter baumannii* complex, and *Pseudomonas aeruginosa* in selected clinical specimens from January 1 to March 31, 2020 was analyzed using the WHONET 5.6 platform. The following clinical isolates recovered from selected specimens were included: carbapenem-resistant *Enterobacteriaceae*, ESBL or carbapenem-resistant *E*. *coli*, and *K*. *pneumoniae*, carbapenem-resistant *A*. *baumannii* complex, and *P*. *aeruginosa*. Strains were genotyped to detect ESBL and/or carbapenemase-encoding genes.

**Results:**

Among blood isolates, *A*. *baumannii* complex showed more than 68% resistance for all antibiotics tested, and among Enterobacteria, *E*. *cloacae* complex showed higher resistance to carbapenems. *A*. *baumannii* complex showed a higher resistance pattern for respiratory specimens, with only amikacin having a resistance lower than 70%. Among *K*. *pneumoniae* isolates, *bla*_TEM_, *bla*_SHV_, and *bla*_CTX_ were detected in 68.79%, 72.3%, and 91.9% of isolates, respectively. Among *E*. *coli* isolates, *bla*_TEM_, *bla*_SHV_, and *bla*_CTX_ were detected in 20.8%, 4.53%, and 85.7% isolates, respectively. For both species, the most frequent genotype was *bla*_CTX-M-15_. Among *Enterobacteriaceae*, the most frequently detected carbapenemase-encoding gene was *bla*_NDM-1_ (81.5%), followed by *bla*_OXA-232_ (14.8%) and *bla*_oxa-181_(7.4%), in *A*. *baumannii* was *bla*_OXA-24_ (76%) and in *P*. *aeruginosa*, was *bla*_IMP_ (25.3%), followed by *bla*_GES_ and *bla*_VIM_ (13.1% each).

**Conclusion:**

Our study reports that NDM-1 is the most frequent carbapenemase-encoding gene in Mexico in Enterobacteriaceae with the circulation of the oxacillinase genes 181 and 232. KPC, in contrast to other countries in Latin America and the USA, is a rare occurrence. Additionally, a high circulation of ESBL *bla*_CTX-M-15_ exists in both *E*. *coli* and *K*. *pneumoniae*.

## Introduction

National and local surveillance of drug resistance and the involved genotypes is fundamental to implementing adequate infection control measures [1, 2].

The prevalence of carbapenemases from Ambler class A, B, and D, cephalosporinases (AmpCs), is rapidly increasing among Gram-negative bacteria and is rapidly increasing among Gram-negative bacteria and is now widely distributed [3, 4].

Among class A, the most reported β-lactamases are the extended-spectrum β-lactamases (ESBLs) cefotaximase (CTX-M), temoneira (TEM), and sulfhydryl variable (SHV), along with the *Klebsiella pneumoniae* carbapenemase (KPC) [3, 4].

Class B metallo-β-lactamases include those enzymes that confer resistance to carbapenem antibiotics as the carbapenemases the imipenem (IMP), New Delhi metallo-β-lactamase (NDM), and those encoded by vimentin (VIM) [5]. Among class D β-lactamases, the most frequently reported oxacillinases (OXA) are those encoded by *bla*_OXA-23-like_, *bla*_OXA-24-like_, and *bla*_OXA-58-like_ genes in *Acinetobacter baumannii* and by *bla*_OXA-48-like_, especially in *Enterobacteriaceae*.

Some research groups from Mexico have published the drug resistance rates and involved genes for some Gram-negative bacteria, including *A*. *baumannii*, *Pseudomonas aeruginosa*, *Enterobacter cloacae*, *K*. *pneumoniae*, and *Escherichia coli* [6–9]. However, information available is limited, and nationwide studies are needed.

To contribute to the study of drug resistance in Mexico, the Network for the Research and Surveillance of Drug Resistance (*Red Temática de Investigación y Vigilancia de la Farmacorresistencia* INVIFAR, in Spanish) was created in 2018 and has reported an increase in drug resistance for several bacterial species, underlying the increase in carbapenem resistance for *Enterobacter* spp. and *Klebsiella* spp. [10, 11].

This report presents phenotypic and genetic data on the prevalence and characteristics of ESBL and carbapenemase-producing representative Gram-negative species in Mexico during the first trimester of 2020.

## Materials and methods

### Participating centers, data collection, and analysis

A total of 52 centers participated: 43 hospital-based laboratories and 9 external laboratories.

Identification and susceptibility test results from January 1 to March 31, 2020, from participating laboratories were deposited into the WHONET 5.6 platform and converted to the WHONET using the BacLink 2 tool. WHONET files were analyzed using macros to facilitate the revision, and only one strain per patient was included. The distribution of antimicrobial resistance for *E*. *coli*, *K*. *pneumoniae*, *E*. *cloacae* complex, *A*. *baumannii* complex, and *P*. *aeruginosa* was analyzed in clinical specimens such as urine, blood, and respiratory specimens. The results were scored according to the Clinical and Laboratory Standards Institute (CLSI) criteria in all laboratories [12].

### Included isolates

Participating laboratories sent to the coordinating laboratory all recovered isolates with the following characteristics: carbapenem-resistant *Enterobacteriaceae* (any species); ESBL or carbapenem-resistant *E*. *coli* collected from urine or blood; ESBL or carbapenem-resistant *K*. *pneumoniae* recovered from urine, respiratory specimens (endotracheal and bronchoalveolar lavage), or blood; carbapenem-resistant *A*. *baumannii* complex and *P*. *aeruginosa* recovered from urine, respiratory specimens, or blood. Clinical isolates collected from January 1 to March 31, 2020, were included.

All identifications were confirmed at the coordinating laboratory using MALDI-TOF. After confirmation, phenotypic tests and genotyping tests were performed for each strain.

### Beta-lactamase identification and characterization in *Enterobacteriaceae*

The ESBL phenotypic detection test was performed using the double disk method recommended by the CLSI for *E*. *coli* and *K*. *pneumonia* [12]. The molecular detection and characterization of ESBLs were performed for *bla*_TEM_, *bla*_SHV_, and *bla*_CTX-M_ genes in selected isolates by PCR using previously described and newly designed primers (S1 Table) [13]. A selection of amplified products was sequenced.

Carbapenemase production in *Enterobacteriaceae* was detected using the CarbaNP test and modified carbapenem inactivation according to the CLSI [12].

For carbapenemase-encoding genes detection, *Enterobacteriaceae* were tested by PCR for *bla*_KPC_, *bla*_GES_, *bla*_VIM_, *bla*_IMP_, *bla*_NDM-1_, *bla*_OXA-48-like_, and chromosomal *ampC* genes as described [14–17].

All PCR products were sequenced using a Hitachi analyzer (Applied Biosystems, Hitachi High-Technologies Corporation, Tokyo, Japan). DNA sequences were aligned and edited using BioEdit software (Ibis Bioscience, Carlsbad, CA) and matched in a gene bank (www.ncbi.nlm.nih.gov/genbank).

### Carbapenemase assays in *A*. *baumannii* and *P*. *aeruginosa*

For carbapenem-resistant *A*. *baumannii*, the *bla*_OXA-23_, *bla*_OXA-24_, *bla*_OXA-51_, *bla*_OXA-58_, *bla*_VIM_, *bla*_IMP_, and *bla*_NDM-type_ β-lactamase genes were screened using PCR as described elsewhere [18, 19]. For *P*. *aeruginosa*, the detection of carbapenemase-encoding genes *bla*_KPC_, *bla*_GES_, *bla*_IMP_, *bla*_NDM_, and *bla*_VIM_ was performed by PCR as described previously [20–24].

### Ethics statement

The local ethics committee of Hospital Civil de Guadalajara “Fray Antonio Alcalde,” Jalisco, Mexico) approved this study with reference number 129/17. Informed consent was waived by the ethics committee because no intervention was involved. All participating institutions agreed with the present study.

## Results

### Participating centers, data, and collected strains

In this study, 52 centers collected strains and sent them to the coordinating laboratory: 43 hospital-based laboratories and 9 external laboratories. The three-month identification and susceptibility data were obtained from 46 centers (37 hospital-based laboratories and 9 external laboratories). The centers were distributed across 19 Mexican states. The characteristics of hospital-based centers are shown in Table 1.

**Table 1:** Characteristics of hospitals participating.

Center	Pr/Pu	Type	Hosp beds	ICU Beds
Hospital General con Especialidades Juan María de Salvatierra	Pu	Spe	120	18
Bioclinsa, Hospital Ginequito	Pu	M&Ch	93	26
Centenario Hospital Miguel Hidalgo	Pu	Pu	103	21
Galenia Hospital	Pr	Spe	54	4
Hospital Ángeles Morelia	Pr	Spe	50	11
Hospital Clínica Nova	Pr	Spe	44	4
Hospital de Alta Especialidad de Veracruz	Pu	Spe	235	10
Hospital de Especialidades Pediátricas de Chiapas	Pu	Ped	90	19
Hospital General Ciudad Obregón	Pu	Univ	156	5
Hospital H+ Querétaro	Pr	Spe	33	5
Hospital Infantil de Morelia “Eva Sámano de López Mateos”	Pu	Ped	80	6
Hospital Regional de Alta Especialidad del Bajío	Pu	Spe	184	29
Hospital Regional Delicias	Pu	Spe	67	8
Hospital Regional Universitario de Colima	Pu	Univ	108	8
Hospital Ángeles Valle Oriente	Pr	Spe	71	21
Hospital Civil de Guadalajara “Fray Antonio Alcalde”	Pu	Univ	1000	85
Hospital de Especialidad Materno Infantil de León	Pu	M&Ch	16	70
Hospital de Especialidades Pediátricas León	Pu	Ped	38	17
Hospital de la Madre y Niño Guerrerense	Pu	M&Ch	30	10
Hospital General “Dr. Manuel Gea González”	Pu	Gen	107	5
Hospital General "Dr. Miguel Silva"	Pu	Gen	300	14
Hospital General “Dr. Raymundo Abarca Alarcón”	Pu	Gen	108	8
Hospital General de Chetumal	Pu	Gen	88	10
Hospital General de Chilpancingo	Pu	Gen	114	8
Hospital General de Zona No 21	Pu	Gen	73	9
Hospital General del Estado “Dr. Ernesto Ramos Bours”	Pu	Univ	200	20
Hospital General Regional No.1	Pu	Gen	233	10
Hospital General de Zona No.1	Pu	Gen	180	22
Hospital General de Zona No 1 “Dr. Demetrio Mayoral Pardo”	Pu	Gen	168	8
Hospital Materno Infantil "Morelos"	Pu	M&Ch	30	0
Hospital Militar Regional de Especialidades de Mazatlán	Pu	Spe	126	6
Hospital para el Niño Poblano	Pu	Ped	90	17
Hospital Regional Bicentenario de la Independencia, ISSSTE	Pu	Spe	206	8
Hospital Regional de Alta Especialidad de Oaxaca	Pu	Spe	60	6
Hospital Regional Monterrey ISSSTE Monterrey	Pu	Spe	141	25
Hospital Universitario "Dr. José Eleuterio González"	Pu	Univ	670	46
Instituto Materno infantil del Estado de México	Pu	M&Ch	115	30
Instituto Nacional de Cancerología	Pu	Spe	135	6
Instituto Nacional de Cardiología “Ignacio Chávez”	Pu	Spe	249	28
Instituto Nacional de Ciencias Médicas y Nutrición “Salvador Zubirán”	Pu	Spe	170	14
Instituto Nacional de Rehabilitación “Luis Guillermo Ibarra Ibarra”	Pu	Spe	228	20
Sanatorio La Luz	Pr	Gen	30	3
Swiss Hospital	Pr	Pr	Spe	55

Abbreviations: Ad, adults; Gen, general; M&Ch, mother and child; Pr, Private, Pu, Public; Ped, pediatrics; Spe, specialties; Univ.

The results of drug susceptibility for 8,245 strains were included for analysis, and 2,243 clinical isolates were collected at the reference laboratory. A selection of 813 isolates (including isolates from each center and state) was included for phenotypic and genotypic analysis.

### Drug resistance

Regarding urine isolates, resistance was higher than 55% for all antibiotics in *A*. *baumannii* complex. In *P*. *aeruginosa*, the lowest percentage of resistance was for piperacillin/tazobactam (29.5%). Meanwhile, carbapenem resistance was low in *E*. *coli* (<1%) but high in *E*. *cloacae* complex (10.9%) (Table 2). Also, 44.9% and 39.3% of *E*. *coli* and *K*. *pneumoniae*, respectively, were reported to be ESBLs producers.

**Table 2:** Percentage of resistant, intermediate, and susceptible gram-negative isolates collected from urine.

	*A*. *baumannii* complex				*P*. *aeruginosa*				*K*. *pneumoniae*				*E*. *coli*				*E*. *cloacae complex*			
Antibiotic	n	%R	%I	%S	n	%R	%I	%S	n	%R	%I	%S	n	%R	%I	%S	n	%R	%I	%S
AMK	ND	ND	ND	ND	151	31.8	4.0	64.2	306	2.0	0.0	98.0	2215	3.0	0.7	96.3	49	20.4	0.0	79.6
AMC	ND	ND	ND	ND	ND	ND	ND	ND	ND	ND	ND	ND	462	11.7	14.3	74.0	ND	ND	ND	ND
AMP	ND	ND	ND	ND	ND	ND	ND	ND	ND	ND	ND	ND	2998	75.6	1.1	23.3	ND	ND	ND	ND
AZT	ND	ND	ND	ND	ND	ND	ND	ND	43	44.2	0.0	55.8	201	65.2	0.5	34.3	ND	ND	ND	ND
CAZ	41	87.8	0.0	12.2	148	33.8	6.1	60.1	301	46.5	0.3	53.2	2220	45.9	0.4	53.7	49	38.8	0.0	61.2
FEP	47	85.1	0.0	14.9	207	31.9	1.9	66.2	431	42.9	0.0	57.1	3017	43.3	0.6	56.1	55	29.1	1.8	69.1
FOX	ND	ND	ND	ND	ND	ND	ND	ND	85	45.9	1.2	52.9	999	41.3	1.1	57.6	ND	ND	ND	ND
CIP	63	82.5	0.0	17.5	230	46.5	1.3	52.2	530	40.4	5.8	53.8	3449	60.7	1.7	37.6	64	21.9	6.2	71.9
CTX	36	58.3	8.3	33.4	ND	ND	ND	ND	440	44.3	0.5	55.2	2494	46.3	0.1	53.6	47	38.3	0.0	61.7
GEN	65	63.1	4.6	32.3	234	30.8	10.3	58.9	548	33.9	0.7	65.4	3551	31.3	1.0	67.7	67	25.4	1.5	73.1
IMP	ND	ND	ND	ND	50	44.0	2.0	54.0	97	1.0	1.0	98.0	824	0.8	0.1	99.1	17	5.9	0.0	94.1
LVX	ND	ND	ND	ND	ND	ND	ND	ND	77	22.1	2.6	75.3	566	46.3	0.7	53.0	12	16.7	0.0	83.3
MEM	41	82.9	0.0	17.1	205	38.5	5.9	55.6	422	2.1	0.5	97.4	2999	0.7	0.1	99.2	55	10.9	3.6	85.5
NIT	ND	ND	ND	ND	ND	ND	ND	ND	389	32.6	30.6	36.8	2381	8.1	4.8	87.1	56	37.5	30.4	32.1
NOR	ND	ND	ND	ND	111	39.6	4.5	55.9	235	24.7	3.4	71.9	1694	54.5	1.5	44.0	36	25.0	5.6	69.4
SAM	ND	ND	ND	ND	ND	ND	ND	ND	299	49.5	10.0	40.5	2135	43.2	20.7	36.1	ND	ND	ND	ND
TZP	28	92.9	0.0	7.1	73	20.5	20.5	59.0	125	8.0	10.4	81.6	1212	6.4	7.2	86.4	24	25.0	4.2	70.8
SXT	39	69.2	0.0	30.8	ND	ND	ND	ND	384	53.6	0.0	46.4	2532	55.8	0.0	44.2	56	35.7	0.0	64.3

Abbreviations: AMC, amoxicillin-clavulanic acid; AMK, amikacin; AMP, ampicillin; AZT, aztreonam; CAZ, ceftazidime; CIP, ciprofloxacin; CTX, cefotaxime; FEP, cefepime; FOX, cefoxitin; GEN, gentamicin; IMP, imipenem; LVX, levofloxacin; MEM, meropenem; NIT, nitrofurantoin; NOR, norfloxacin; SAM, ampicillin-sulbactam; SXT, trimethoprim-sulfamethoxazole; TZP, piperacillin-tazobactam. R, resistant; I, intermediate; S, susceptible. ND, Not determined.

Among blood isolates, *A*. *baumannii* showed more than 68% resistance for all antibiotics tested, and *P*. *aeruginosa* had 37.1% resistance to meropenem. Among *Enterobacteriaceae*, *E*. *cloacae* showed higher resistance to carbapenems (4.4% for meropenem), whereas *K*. *pneumoniae* and *E*. *coli* had more than 59% resistance for cefepime (Table 3).

**Table 3:** Percentage of resistant, intermediate, and susceptible gram-negative isolates collected from blood.

	*A*. *baumannii* complex				*P*. *aeruginosa*				*K*. *pneumoniae*				*E*. *coli*				*E*. *cloacae*			
Antibiotic	n	%R	%I	%S	n	%R	%I	%S	n	%R	%I	%S	n	%R	%I	%S	n	%R	%I	%S
AMK	20	70.0	5.0	25.0	54	16.7	3.7	79.6	79	2.5	1.3	96.2	136	2.9	0.7	96.4	41	2.4	0.0	97.6
AMP	ND	ND	ND	ND	ND	ND	ND	ND	ND	ND	ND	ND	83	86.7	0.0	13.3	ND	ND	ND	ND
CAZ	73	85.0	1.4	13.6	55	16.4	20.0	63.6	78	64.1	1.3	34.6	135	66.7	0.0	33.3	41	24.4	0.0	75.6
FEP	76	83.0	0.0	17.0	71	15.5	11.3	73.2	102	59.8	0.0	40.2	197	61.9	0.5	37.6	45	11.1	4.4	84.5
FOX	ND	ND	ND	ND	ND	ND	ND	ND	48	62.5	0.0	37.5	83	71.1	1.2	27.7	ND	ND	ND	ND
CIP	90	83.0	0.0	17.0	68	14.7	5.9	79.4	106	32.1	13.2	54.7	180	67.8	0.6	31.6	44	4.5	2.3	93.2
CTX	41	78.0	9.8	12.2	ND	ND	ND	ND	40	65.0	0.0	35.0	55	60.0	1.8	38.2	20	25.0	0.0	75.0
GEN	92	71.0	3.3	25.7	70	12.9	8.6	78.5	113	38.1	0.9	61.0	201	44.3	2.0	53.7	47	21.3	0.0	78.7
IPM	16	69.0	0.0	31.0	26	46.2	0.0	53.8	44	4.5	2.3	93.2	103	1.0	0.0	99.0	15	7.1	0.0	92.9
LVX	17	82.0	0.0	18.0	11	36.4	9.1	54.5	19	15.8	5.3	78.9	26	57.7	0.0	42.3	ND	ND	ND	ND
MEM	75	81.0	1.3	17.7	70	37.1	15.7	47.2	104	2.9	0.0	97.1	197	1.5	0.0	98.5	45	4.4	0.0	95.6
SAM	73	75.0	8.2	16.8	ND	ND	ND	ND	84	52.4	9.5	38.1	169	51.5	14.2	34.3	ND	ND	ND	ND
SXT	47	75.0	0.0	25.0	ND	ND	ND	ND	50	56.0	0.0	44.0	81	63.0	0.0	37.0	23	30.4	0.0	69.6
TZP	48	90.0	0.0	10.0	45	15.6	13.3	71.1	79	10.1	13.9	76.0	144	6.2	6.2	87.6	30	16.7	10.0	73.3

Abbreviations: AMK, amikacin; AMP, ampicillin; CAZ, ceftazidime; CIP, ciprofloxacin; CTX, cefotaxime; FEP, cefepime; FOX, cefoxitin; GEN, gentamicin; IMP, imipenem; LVX, levofloxacin; MEM, meropenem; NIT, nitrofurantoin; SAM, ampicillin/sulbactam; SXT, trimethoprim-sulfamethoxazole; TZP, piperacillin-tazobactam. R, resistant; I, intermediate; S, susceptible. ND, Not determined

Also, 60% and 49.3% of *E*. *coli* and *K*. *pneumoniae*, respectively, were reported to be ESBLs producers.

*A*. *baumannii* showed a higher resistance pattern in respiratory specimens, with only amikacin exhibiting a resistance less than 70%. In general, *K*. *pneumoniae* had higher resistance to antibiotics than *E*. *cloacae* (Table 4). Also, 47% of *K*. *pneumoniae* isolates were reported to be ESBLs producers.

**Table 4:** Percentage of resistant, intermediate, and susceptible gram-negative isolates collected from respiratory specimens.

	*A*. *baumannii complex*				*P*. *aeruginosa*				*K*. *pneumoniae*				*E*. *cloacae*			
Antibiotic	n	%R	%I	%S	n	%R	%I	%S	n	%R	%I	%S	n	%R	%I	%S
AMK	39	69.0	15.0	16.0	105	15.2	4.8	80.0	62	0.0	0.0	100.0	33	0.0	0.0	100.0
AMC	ND	ND	ND	ND	ND	ND	ND	ND	16	18.8	18.8	62.5	ND	ND	ND	ND
AMP	ND	ND	ND	ND	ND	ND	ND	ND	ND	ND	ND	ND	21	90.5	0.0	9.5
CTX	68	79.0	7.4	13.6	ND	ND	ND	ND	43	60.5	0.0	39.5	20	35.0	0.0	55.0
CAZ	130	89.0	1.5	9.5	105	23.8	12.4	63.8	62	48.4	1.6	50.0	33	27.3	0.0	72.7
FEP	174	89.0	1.1	9.9	141	15.6	5.0	79.4	96	46.9	0.0	53.1	58	5.2	3.4	91.4
FOX	ND	ND	ND	ND	ND	ND	ND	ND	31	45.2	0.0	54.8	ND	ND	ND	ND
CIP	160	86.0	0.0	14.0	139	27.3	2.9	69.8	87	31.0	4.6	64.4	42	4.8	2.4	92.8
GEN	163	73.0	7.4	20.0	146	16.4	8.2	75.4	104	34.6	1.0	64.4	57	5.3	0.0	94.7
IPM	62	90.0	0.0	10.0	64	50.0	0.0	50.0	51	0.0	0.0	100.0	38	0.0	7.9	92.1
LVX	65	92.0	0.0	8.0	31	3.2	6.5	90.3	43	20.9	2.3	76.8	25	4.0	0.0	96.0
MEM	163	87.0	0.6	12.4	137	33.6	16.1	50.4	97	2.1	0.0	97.9	59	1.7	0.0	98.3
SAM	131	81.0	5.3	14.0	ND	ND	ND	ND	68	48.5	2.9	48.6	ND	ND	ND	ND
TZP	113	93.0	1.8	5.3	92	21.7	10.9	67.4	80	16.2	7.5	76.3	44	13.6	2.3	84.1
SXT	82	79.0	0.0	21.0	ND	ND	ND	ND	71	50.7	0.0	49.3	40	17.5	0.0	82.5

Abbreviations: AMC, amoxicillin-clavulanic acid; AMK, amikacin; AMP, ampicillin; CAZ, ceftazidime; CIP, ciprofloxacin; CTX, cefotaxime; FEP, cefepime; FOX, cefoxitin; GEN, gentamicin; IMP, imipenem; LVX, levofloxacin; MEM, meropenem; SAM, ampicillin-sulbactam; SXT, trimethoprim-sulfamethoxazole; TZP, piperacillin-tazobactam; TGC, tigecycline; TOB, tobramycin. R, resistant; I, intermediate; S, susceptible.

### ESBL phenotype and genotype

A total of 1059 *E*. *coli* and 370 *K*. *pneumoniae* from selected specimens were received. A selection of isolates was evaluated for further analysis (including representative isolates from each center).

Among isolates selected for analysis, 173/215 *K*. *pneumoniae* and 419/425 *E*. *coli* were confirmed to be ESBLs using the double disk method. All were screened using PCR to detect ESBL-encoding genes *bla*_TEM_, *bla*_SHV_, and *bla*_CTX_.

Among *K*. *pneumoniae* isolates, *bla*_TEM_, *bla*_SHV_, and *bla*_CTX_ were detected in 119/173, 68.8%, 125/173,72.3%, and 159/173, 91.9% of isolates, respectively, with 124/173 (71.7%) isolates carrying both *bla*_SHV_ and *bla*_CTX_. A selection of ESBL PCR products were sequenced and most of the *bla*_CTX-M_ genes were detected to be *bla*_CTX_-_M-15_ (15/17, 88.23%) followed by *bla*_CTX-M-55_ (7/17,41.17%). Among the *bla*_SHV_ gene, a great diversity was detected, including *bla*_SHV-11_, *bla*_SHV-28_, *bla*_SHV-158_, *bla*_SHV-171_, *bla*_SHV-176_, *bla*_SHV-196_, *bla*_SHV-205_, *bla*_SHV-213_, and *bla*_SHV-228_. Some of them with no evidence of ESBL activity (Table 5).

**Table 5:** Distribution of ESBL genotypes among *E*. *coli* and *K*. *pneumoniae* selected isolates.

n	*bla*_TEM-1_	*bla*_SHV_	*bla*_CTX_
*E*. *coli*			
8			*bla*_CTX-M-15_
7			*bla*_CTX-M-15,_ *bla*_CTX-M-55_
7	*bla*_TEM-1_		*bla*_CTX-M-15,_ *bla*_CTX-M-55_
3	*bla*_TEM-1_		*bla*_CTX-M-15_
1	*bla*_TEM-1_	*bla*_SHV-5_	*bla*_CTX-M-15,_ *bla*_CTX-M-55_
1	*bla*_TEM-1_		*bla*_CTX-M-15,_ *bla*_CTX-M-14_
1	*bla*_TEM-1_	*bla*_SHV-11_	*bla*_CTX-M-15_
1	*bla*_TEM-1_		*bla*_CTX-M-15,_ *bla*_CTX-M-177_
1	*bla*_TEM-1_	*bla*_SHV-171_	*bla*_CTX-M-15_
1	*bla*_TEM-1_	*bla*_SHV-38_	*bla*_CTX-M-15,_ *bla*_CTX-M-55_
1	*bla*_TEM-1_		*bla*_CTX-M-15_
1	*bla*_TEM-166_		*bla*_CTX-M-55_
1			*bla*_CTX-M-27_
*K*. *pneumoniae*			
2		*bla*_SHV-213_	*bla*_CTX-M-15,_ *bla*_CTX-M-55_
1	*bla*_TEM-1_	*bla*_SHV-171_	*bla*_CTX-M-15_
1	*bla*_TEM-1_	*bla*_SHV-11_	*bla*_CTX-M-15_
1	*bla*_TEM-1_	*bla*_SHV-228_	*bla*_CTX-M-15,_ *bla*_CTX-M-55_
1	*bla*_TEM-1_	*bla*_SHV-205_	*bla*_CTX-M-15,_ *bla*_CTX-M-55_
1	*bla*_TEM-1_	*bla*_SHV-28_	*bla*_CTX-M-15,_ *bla*_CTX-M-55_
1	*bla*_TEM-1_	*bla*_SHV-158_	*bla*_CTX-M-15_
1	*bla*_TEM-1_		*bla*_CTX-M-15_
1	*bla*_TEM-228_		*bla*_CTX-M-15_
1		*bla*_SHV-171_	
1		*bla*_SHV-176_	*bla*_CTX-M-15_
1		*bla*_SHV-11_	*bla*_CTX-M-15,_ *bla*_CTX-M-55_
1		*bla*_SHV-196_	*bla*_CTX-M-15_
1		*bla*_SHV-11_	*bla*_CTX-M-15,_ *bla*_CTX-M-101_
1		*bla*_SHV-11_	
1			*bla*_CTX-M-15,_ *bla*_CTX-M-55_

Among *E*. *coli* isolates, *bla*_TEM_, *bla*_SHV_, and *bla*_CTX_ were detected in 87/419, 20.76%, 19/419, 4.53%, and 359/419, 85.68% of isolates, respectively, with 18 (4.29%) isolates carrying both *bla*_SHV_ and *bla*_CTX_.

A selection of ESBL PCR products were sequenced and most of the *bla*_CTX-M_ encoding genes were detected to be *bla*_CTX_-_M-15_ (32/34, 94.1%), followed by *bla*_CTX-M-55_ (17/34, 50.0%). Among the *bla*_SHV_ gene, *bla*_SHV-5_ and *bla*_SHV-11_ (with reported ESBL activity), *bla*_SHV-38_ (with reported carbapenemase activity), and *bla*_SHV-171_ (with no report of ESBL activity) were detected (Table 5).

### Carbapenemase-encoding genes

A total of 26 carbapenem-resistant *Enterobacteriaceae* isolates were received for genotyping (one of them with a subpopulation). Carbapenem-encoding genes were detected primarily in *E*. *coli*, followed by *K*. *pneumoniae*. The most frequently detected carbapenemase-encoding gene was *bla*_NDM-1_ (81.5%), followed by *bla*_OXA-232_ (14.8%) and *bla*_oxa-181_(7.4%). One *K*. *pneumoniae* isolate was detected to harbor both *bla*_KPC_ and *bla*_NDM-1_. (Table 6).

**Table 6:** Distribution of carbapenemase-encoding genes among selected carbapenem-resistant Enterobacteriaceae^a^.

Isolate	Specimen	Species	*bla*_KPC-like_	*bla*_OXA-48-like_	*bla*_NDM-1_	*bla*_IMP-2_	*bla*_CTXM-15_	*ampC*
53	Blood	*Enterobacter cloacae*	-	-	+	-	+	-
223	Blood	*Enterobacter cloacae*	-	-	+	-	+	-
255	Blood	*Klebsiella pneumoniae*	-	-	+	-	+	-
303	Blood	*Serratia marcescens*	-	-	+	-	-	-
463	Blood	*Escherichia coli*	-	-	+	+	+	-
489	Catheter	*Klebsiella pneumoniae*	-	-	+	+	+	-
562	Urine	*Providencia rettgeri*	-	-	+	-	-	-
591	Urine	*Klebsiella variicola*	-	-	+	-	+	-
849	Abscess	*Escherichia coli*	-	*bla*_OXA-181_	-	-	-	-
850	BAL	*Escherichia coli*	-	-	+	-	+	-
851	BAL	*Escherichia coli*	-	*bla*_OXA-181_	-	-	+	-
853	Blood	*Escherichia coli*	-	-	+	-	+	-
854	Urine	*Escherichia coli*	-	-	+	-	+	-
861	Wound	*Klebsiella oxytoca*	-	-	+	-	+	-
882	Urine	*Escherichia coli*	-	-	+	-	+	-
891	Urine	*Klebsiella pneumoniae*	*bla*_KPC-2_	-	+	-	-	-
1202	Blood	*Escherichia coli*	-	*bla*_OXA-232_	-	-	-	-
1203	Blood	*Klebsiella pneumoniae*	*bla*_KPC-2_	-	+	-	+	-
1457	BAL	*Escherichia coli*	-	-	+	-	+	-
1627	Urine	*Klebsiella variicola*	-	-	+	+	+	-
2063	No data	*Enterobacter xiangfangensis*	-	-	+	-	+	+
2177	Blood	*Escherichia coli*	-	*bla*_OXA-232_	-	-	+	-
2178	Urine	*Escherichia coli*	-	*bla*_OXA-232_	-	-	-	-
2175–1	Abscess	*Escherichia coli*	-	*bla*_OXA-232_	-	-	-	-
2175–2	Abscess	*Escherichia coli*	-	-	+	-	+	+
1562–1	Blood	*Escherichia coli*	-	-	+	-	+	-
1562–2	Blood	*Escherichia coli*	-	-	+	-	+	-

Abbreviation: BAL, bronchoalveolar lavage.

^a^All strains were negative for *bla*_GES_, *bla*_IMI-1_, *bla*_IMP-1_, and *bla*_VIM_.

A total of 102 carbapenem-resistant *A*. *baumannii* isolates were received, and the most frequent carbapenemase-encoding gene was *bla*_OXA-24_ (76%), followed by *bla*_OXA-23_ (18.5%). Other genes detected were *bla*_VIM_ and *bla*_NDM_ (Table 7). All the isolates were negative to *bla*_KPC_, *bla*_GES_, and *bla*_OXA-58_.

**Table 7:** Distribution of carbapenemase-encoding genes among *A*. *baumannii* complex^a^.

n	*bla*_OXA23_	*bla*_OXA24_	*bla*_VIM_	*bla*_NDM_
66	-	+	-	-
15	+	-	-	-
9	-	+	+	-
4	-	-	+	-
3	+	+	-	-
3	-	-	-	-
1	-	-	+	+
1	+	-	+	-

^a^ All strains were negative for *bla*_KPC_ and *bla*_GES_ and positive for *bla*_OXA51_

Regarding carbapenem-resistant *P*. *aeruginosa*, 93 isolates were received, and the carbapenemase-encoding genes most frequently detected were *bla*_IMP_ (25.3%), *bla*_GES_, and *bla*_VIM_ (13.1% each), with 44 (47.31%) isolates containing none of the screened carbapenemase-encoding genes (Table 8).

**Table 8:** Distribution of carbapenemase-encoding genes among *P*. *aeruginosa* carbapenem-resistant clinical isolates *.

n	Specimen	*bla*_GES_	*bla*_VIM_	*bla*_IMP_
24	Respiratory	-	-	-
10	Blood	-	-	-
10	Urine	-	-	-
23	Urine	-	-	+
5	Urine	+	-	-
5	Respiratory	+	-	-
4	Blood	-	+	-
4	Urine	-	+	-
3	Respiratory	-	+	-
2	Blood	+	-	-
1	Urine	-	+	+
1	Blood	-	-	+
1	Urine	+	+	-

^an^ All strains were negative for *bla*_KPC_ and *bla*_NDM_

## Discussion

This report presents phenotypic and genetic data on the frequency and characteristics of ESBL and representative carbapenemase-producing Gram-negative species in Mexico using strains collected from 52 centers in 19 Mexican states.

OXA-48-like carbapenemases are important causes of carbapenem resistance and are now the most common carbapenemase in some populations [25]. In *Enterobacteriaceae*, several variants of *bla*_OXA-48_ have been identified, with *bla*_OXA-181_ and *bla*_OXA-232_ being the two most common [26, 27]. Kinetic properties of these two enzymes had been measured, and both appear broadly similar to *bla*_OXA-48_ in their activity, with *bla*_OXA-232_ demonstrating better hydrolysis of penicillin [28]. In this study, *bla*_OXA-181_ and *bla*_OXA-232_ were detected in *E*. *coli*. At present, *bla*_OXA-232_ has been reported in Mexico in two single-center reports: in *E*. *coli*, carrying *bla*_OXA-232_ plus *bla*_CTXM-15_ [8] and in a case-control-control study in which the infection by *bla*_OXA-232_ strains was associated with the previous use of β-lactam/β-lactamase antibiotics (OR, 6.2) [29]. The OXA-181 variant has been associated with other carbapenemase genes, including *bla*_NDM-1_ and *bla*_VIM-5_ [30]. No previous reports of *bla*_OXA-181_ circulation in Mexico were identified in the literature.

*Enterobacteriaceae*-producing OXA-48-like enzymes are rapidly spreading, and thus, laboratory detection should be optimized. This enzyme has low-level hydrolytic activity against carbapenems and, thus, may not be detected [27]. As detected in this study, *bla*_OXA-48-like_ genes can co-harbor genes encoding ESBL or AmpC enzymes, or both, which confers nonsusceptibility to aztreonam, extended-spectrum cephalosporins, and carbapenem agents and renders these genes a serious menace [31].

NDM has a worldwide distribution, with multiple reports in Asia and Europe since this enzyme was first described in 2007 [32–37]. However, it has remained uncommon in *Enterobacteriaceae* in America, with some reports in Canada, the United States, and Latin American countries [38–41]. In this study, the most frequently detected carbapenemase-encoding gene was *bla*_NDM-1_. The NDM carbapenemase was first described in Mexico in 2013 [6], and since then, several reports have been published about it in the county [8, 41]. According to our report, NDM is now the most prevalent carbapenemase in Mexico. This study reports by Mexico the first NDM-1-positive *Klebsiella variicola* isolates considered an emerging pathogen in humans [42].

Within a few years, KPC producers became global as they were reported in America, Europe, and Asia [32, 43]. Interestingly, this enzyme has a lower frequency in Mexico when compared to other Latin American countries, as confirmed by our report [43]. KPC and NDM have received special attention due to limited therapeutic options and high mortality associated with infections caused by strains carrying genes that encode these enzymes [44].

*A*. *baumannii* isolates have resistance rates greater than 50.0% to carbapenems worldwide, and our results confirmed this resistance [45, 46]. In this study, we detected that the most frequent carbapenemase-encoding gene was *bla*_OXA-24_, followed by *bla*_OXA-23_. OXA-23 isolates have been primarily detected in Asia, Europe, the United States, Brazil, and South America, whereas OXA-24 has been reported in Europe, Asia, and North America [5, 47–51].

Among *P*. *aeruginosa* isolates, 44 out of 93 isolates did not contain any of the screened carbapenemase-encoding genes. The most frequent carbapenem resistance mechanism described in *P*. *aeruginosa* is the overexpression of efflux pumps and the loss of the *Opr* porin [52]. Less frequently, genes encoding carbapenemases have been described as an alternative mechanism, with GES variants and IMP, VIM, and NDM reported. In this study, we did not analyze the overexpression of efflux pumps and porins, but *bla*_GES_, *bla*_VIM_, and *bla*_IMP_ genes were detected in approximately half of the strains (49/93) (Table 8). Similar results were reported in Mexico with a prevalence of 36.2% of carbapenemases (IMP, VIM, and GES types) on *P*. *aeruginosa* clinical isolates. These genes have been reported to be chromosomally encoded on embedded class 1 integron arrays [53].

Besides carbapenemase-encoding genes, other important mechanisms conferring carbapenem resistance have been observed, including carbapenem hydrolysis by AmpCs in combination with ESBL enzymes, rendering carbapenem resistance to Gram-negative bacteria [54]. In our study, a high frequency of ESBL-producing *Enterobacteriaceae* was identified, with the AMPc-encoding gene detected in two strains (*Enterobacter xiangfangensis* (a member of the *E*. *cloacae* complex) and *E*. *coli* harboring both *bla*_NDM-1_, *bla*_CTXM-15_, and *ampC*). The presence of AmpC/ESBL and the exact changes of the porins may significantly affect carbapenem resistance. Thus, these mechanisms need to be considered in future research.

The prevalence of bacterial isolates expressing the ESBL phenotype varies across different geographical regions, with rates from 10% to 58% [55]. ESBLs arise primarily due to mutations in the *bla*_TEM_, *bla*_SHV_, or *bla*_CTX_ genes, and at present, the CTX-M type is known to be the most frequent non-TEM, non-SHV ESBL [55]. In our study, 72.25% of ESBL-producing *K*. *pneumoniae* isolates and 85.7% of *E coli* isolates harbored *bla*_CTX-M_, confirming the spread of this enzyme.

The presence of CTX-M-type enzymes is relevant because they are readily inhibited by all commercially available β-lactamase inhibitors, including avibactam, vaborbactam, and relebactam [56]; a valuable alternative therapy to the recommended ertapenem regimen.

In this study, the non-ESBL TEM-1 was frequently detected, and SHV was detected with no predominance of any subtype. Worldwide, the prevalence of TEM and SHV has diminished, mirroring the worldwide dissemination of isolates producing CTX-M-type -lactamases [57].

Some of the limitations of this study are that not all states in Mexico participated, and the analysis of porins was not included. Furthermore, we only included some bacterial species involved in ESBL production. Our network will continue to actively survey drug resistance and molecular mechanisms involved.

In conclusion, our report identifies NDM as the most frequent carbapenemase-encoding gene in *Enterobacteriaceae* Mexico with circulation of the oxacillinase genes 181 and 232. KPC, in contrast to other countries in Latin America and the USA, is a rare occurrence. Additionally, a high circulation of ESBL *bla*_CTX-M-15_ existed in *E*. *coli* and *K*. *pneumoniae*.

## Supporting information

S1 Table Primers used for genotyping of ESLs genes. (DOCX)
