# Supplementary material for: Drug resistance phenotypes and genotypes in Mexico in representative gram-negative species: Results from the infivar network
Source: PLoS One. 2021 Mar 17;16(3):e0248614. doi: 10.1371/journal.pone.0248614 (PMC7968647; doi:10.1371/journal.pone.0248614)
Supplement: S1 Table — (DOCX) [file pone.0248614.s001.docx]

S1 Table. Primers used to ESBL amplification

| Gene | Primer | Sequence (5´-3´) | Product (bp) | Tm  (°C) | Reference |
| --- | --- | --- | --- | --- | --- |
| CTX-M group 1 and 8 | CTX-M 1 and 8 F | TGTGCAGYACCAGTAARGYKATG | 583 | 55 | This study |
|  | CTX-M 1 and 8 R | TARRTSACCAGAAYVAGCGGC |  |  |  |
| CTX-M group 2 | CTX-M 2 F | CGATGTGCAGTACCAGTAAGG | 540 |  | This study |
|  | CTX-M 2 R | CGATATCGTTGGTGGTGC |  |  |  |
| CTX-M group 9 | CTX-M 9 F | ATGGTGACAAAGAGAGTGCAA | 747 |  | This study |
|  | CTX-M 9 R | AATATCATTGGTGGTGCCGTAG |  |  |  |
| CTX-M 151 | CTX-M 151 F | GCGGCCATGATAGGTACG | 786 |  |  |
|  | CTX-M 151 R | AAAGTAAGTCACAATAACCAGCG |  |  |  |
| TEM | TEM F | CAACATTTTCGTGTCGCCC | 844 |  | Arlet G et al. |
|  | TEM R | GCTTAATCAGTGAGGCACC |  |  |  |
| SHV | SHV F | TATTATCTCCCTGTTAGCCA | 783 | 58 | This study |
|  | SHV R | CGCTCTGCTTTGTTATTC |  |  |  |
